# Clinical and pathological factors associated with eGFR slope in IgA nephropathy: a single-center retrospective cohort study

**DOI:** 10.1080/0886022X.2026.2732704

**Published:** 2026-10-07

**Authors:** Liyu Lin, Yiqi Su, Bing Yu, Ziyan Shen, Ying Huang, Caifeng Li, Jie Teng

**Affiliations:** ^a^ Department of Nephrology, Zhongshan Hospital (Xiamen), Fudan University, Xiamen, Fujian, China; ^b^ Nephrology Clinical Quality Control Center of Xiamen, Xiamen, Fujian, China; ^c^ Department of Nephrology, Zhongshan Hospital, Fudan University, Shanghai, Shanghai, China

**Keywords:** IgA nephropathy, eGFR slope, chronic kidney disease progression, tubular atrophy, proteinuria

## Abstract

IgA nephropathy (IgAN) has a heterogeneous clinical course. We retrospectively studied 180 patients with biopsy-proven IgAN and at least 3 years of clinical follow-up. Patient-specific eGFR slopes were estimated from available measurements at biopsy and the 6-, 12-, 18-, 24-, 30-, and 36-month follow-up points. We built a *post hoc* multivariable model guided by clinical relevance. Oxford T was treated as categorical, with T0 as reference. The median eGFR slope was −1.12 mL/min/1.73 m^2^/year, and 35 patients (19.4%) met the operational rapid-progression threshold of < −5 mL/min/1.73 m^2^/year. In the primary model, higher ln(UACR + 1) predicted a steeper decline (*β*= −0.612, 95%CI −1.073 to −0.150). T2 lesions predicted a 3.87 mL/min/1.73 m^2^/year faster loss than T0 (*β*= −3.870, 95%CI −7.087 to −0.654), whereas T1 did not differ from T0. The model *R*^2^ was 0.159 (adjusted *R*^2^=0.130). UACR and T2 estimates remained negative under HC3 inference and after excluding influential observations, although T2 was less stable in some restricted analyses. Baseline albuminuria was the most consistent adverse marker of eGFR slope. T2 lesions were linked to faster decline as well, but this estimate derived from only 16 patients (8.9%) and lost statistical significance when analysis was restricted to ≥5 measurements, making the finding tentative and strictly hypothesis-generating.

## Introduction

IgA nephropathy (IgAN) is the most common primary glomerular disease worldwide and a leading cause of end-stage kidney disease (ESKD) [1,2]. Its clinical course is heterogeneous: kidney function may remain stable for prolonged periods in some patients, whereas others experience rapid decline and kidney failure [3]. Identifying reproducible progression markers would help risk stratification and guide the design of future prospective studies.

Renal failure and a doubling of serum creatinine levels are hard endpoints that take several years to become measurable. The eGFR slope captures renal function decline continuously and has gained recognition as a surrogate endpoint in CKD research [4–6]. Steeper negative slopes mean faster kidney function decline. Following KDIGO 2012, we set the rapid progression threshold at eGFR slope < −5 mL/min/1.73 m^2^/year [7]. Prior work has identified proteinuria, hypertension, and histological severity as key determinants of IgAN progression [8–10]. Although the Oxford Classification (MEST-C) standardizes pathological reporting, the relative prognostic weight of its individual components differs from one cohort to another [11]. Whether clinical variables, histological lesions, and treatment jointly affect eGFR slope in routine Chinese practice is still largely unknown [12].

We included 180 biopsy-proven IgAN patients with at least 3 years of follow-up. Our first aim was to describe how eGFR slopes were distributed. We then explored clinical and pathological associations with decline, compared alternative specifications for Oxford T and Lee grade, and tested robustness in sensitivity analyses. Treatment effects and rapid-progression discrimination were addressed only secondarily, as exploratory work.

## Methods

### Study design and population

This single-center retrospective cohort study was conducted at the Department of Nephrology, Zhongshan Hospital (Xiamen), Fudan University. We screened patients who underwent native kidney biopsy between September 2018 and April 2023. Inclusion criteria were biopsy-proven primary IgAN, age ≥14 years at biopsy, clinical follow-up for at least 3 years, at least three valid eGFR measurements among baseline and the nominal 6-, 12-, 18-, 24-, 30-, and 36-month assessments, and baseline eGFR ≥15 mL/min/1.73 m^2^ (Supplementary Table S1). Exclusion criteria were secondary IgAN, another primary glomerular disease, missing critical baseline pathology data, or initiation of maintenance dialysis or kidney transplantation within 3 years after biopsy because sufficient serial eGFR data for the planned slope analysis were unavailable.

The study followed the Declaration of Helsinki and was approved by the Ethics Committee of Zhongshan Hospital (Xiamen), Fudan University (B2023-134R). Informed consent was waived because of the retrospective design. Data were de-identified before analysis.

### Data collection

Baseline clinical data were extracted within 1 month before or at renal biopsy, including demographics, blood pressure, comorbidities, medication history, serum creatinine, blood urea nitrogen, uric acid, cystatin C, albumin, complete blood count, immunoglobulins, complements, lipids, 24-h urine protein, and urine albumin-to-creatinine ratio (UACR). Both UACR and 24-h urine protein were available for all 180 patients. eGFR values were extracted from the hospital laboratory records and were based on the CKD-EPI 2009 creatinine equation [13]. Two renal pathologists, blinded to outcomes, independently assessed Oxford MEST-C and Lee grade; disagreements were resolved by consensus [14,15]. Treatment initiated within 6 months after biopsy included RAAS blockade, corticosteroids, immunosuppressants, SGLT2 inhibitors, and Chinese patent medicines.

### Outcome definition

The primary outcome was eGFR slope (mL/min/1.73 m^2^/year), estimated separately for each patient by ordinary least squares regression of all valid eGFR values against nominal time from biopsy (0, 0.5, 1.0, 1.5, 2.0, 2.5, and 3.0 years). Values recorded during documented acute kidney injury episodes had been excluded before slope calculation. A negative slope indicates decline; a negative regression coefficient indicates an association with faster decline. The patient-level slope was a pragmatic summary and did not assume that every trajectory was biologically linear. Rapid progression was operationally defined as a slope < −5 mL/min/1.73 m^2^/year [7].

We used patient-specific OLS slopes rather than a linear mixed-effects model (LMM) for practical reasons. Exact visit dates were stripped from the de-identified dataset, so a conventional LMM could not be fitted. More importantly, individual slopes give clinically meaningful summaries that nephrologists are familiar with; they also allowed us to link baseline pathology and clinical variables directly to progression speed. The stability of these slope estimates was checked by bootstrapping and sensitivity analyses. The secondary outcome was the proportion of rapid progressors.

### Statistical analysis

We summarized continuous variables as mean ± SD or median (IQR), and categorical variables as *n* (%). We compared groups using *t* tests, Mann–Whitney *U*, Kruskal–Wallis, chi-square, or Fisher exact tests as appropriate.

We first screened unadjusted associations with eGFR slope using univariable linear regression. For the primary multivariable model, we selected variables *post hoc* based on prior evidence, clinical relevance, and complete data availability, instead of using automated stepwise selection. The model included baseline eGFR, ln(UACR + 1), hematuria, blood WBC count, and categorical Oxford T (T0 as reference, with separate indicators for T1 and T2). These variables capture baseline kidney function, albuminuria, urinary activity, systemic inflammation, and chronic tubulointerstitial damage. We natural-log-transformed UACR and 24-h urinary protein. Lee grade was dropped from the primary model given its strong overlap with Oxford T. Treatment variables were also omitted because their post-biopsy timing makes causal inference impossible.

We checked multicollinearity with variance inflation factors, inspected residuals for heteroscedasticity and outliers, and evaluated influential observations using Cook’s distance. We then performed patient-level bootstrap resampling (2,000 iterations) to refit the fixed primary specification. From this we derived percentile intervals, sign-consistency rates, and optimism-corrected *R*^2^. This procedure tested the stability of the fixed model, not the *post hoc* selection process itself.

For robustness, we applied HC3 heteroscedasticity-consistent standard errors, weighted patients by their number of eGFR measurements, and restricted the cohort to those with at least five measurements. We also refitted the model after dropping baseline eGFR, after removing influential observations (Cook’s distance >4/*n*), and after excluding immunosuppressant users. Alternative pathology specifications entered Oxford T as ordinal, as binary (T1/T2 versus T0), or replaced it with Lee IV–V. All primary-model variables were completely observed, and we did not impute missing follow-up eGFR values.

We also explored discrimination of rapid progression using ridge logistic regression (L2 penalty, *C* = 1.0) and ROC curves, with all predictors standardized. The clinical model contained age, baseline eGFR, ln(UACR + 1), and hematuria. The pathological model added MEST-C and Lee IV–V. The combined model pooled both sets of variables, and the full model further added RAAS inhibitor, glucocorticoid, immunosuppressant, and SGLT2 inhibitor use. We derived stratified bootstrap confidence intervals and optimism-corrected AUCs. With only 35 rapid-progression events, these results are presented only in the Supplementary Material; we do not propose clinical cutoffs or claim incremental discrimination.

All analyses were done in Python 3.12. A two-tailed *p* < .05 served as the threshold for statistical significance. Sensitivity and supplementary analyses were interpreted without multiplicity adjustment.

## Results

### Baseline characteristics

We screened 272 patients and included 180 in the final analysis (Figure 1A). Baseline characteristics are summarized in Table 1. Their median age was 38.0 years (IQR 30.0–49.0), and 107 (59.4%) were male. Median baseline eGFR was 76.0 mL/min/1.73 m^2^ (IQR 51.2–98.0), and median 24-h proteinuria was 0.83 g/24 h (IQR 0.40–1.72).

**Figure 1.**
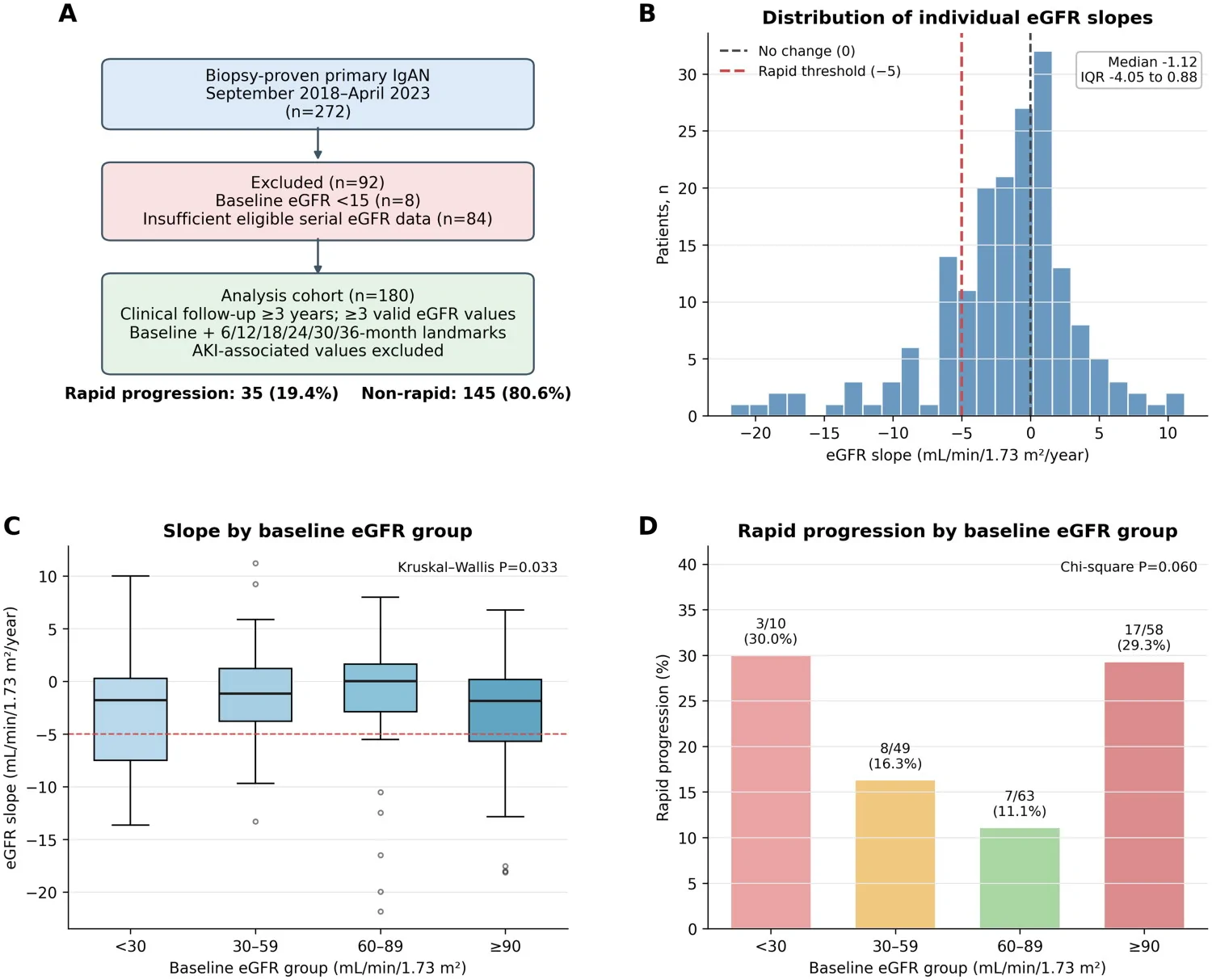
Study design and baseline eGFR-slope characteristics. (A) Patient selection and the nominal slope-estimation schedule; eGFR values recorded during documented acute kidney injury were excluded. (B) Distribution of individual eGFR slopes with reference lines at 0 and −5 mL/min/1.73 m^2^/year. (C) eGFR slope by baseline eGFR group (Kruskal–Wallis *p* = .033). (D). Rapid-progression proportion by baseline eGFR group (chi-square test, *p* = .060); the <30 group included only 10 patients, so the comparison is descriptive and the *p* value should be interpreted cautiously.

**Table 1.** Baseline clinical and pathological characteristics.

Variable	Overall (*n* = 180)	Non-rapid progression (*n* = 145)	Rapid progression (*n* = 35)	*p* Value
Age, years	38.00 (30.00, 49.00)	38.00 (30.00, 49.00)	38.00 (29.50, 49.50)	.728
Male sex	107 (59.4%)	90 (62.1%)	17 (48.6%)	.205
BMI, kg/m²	23.50 (21.42, 25.56)	23.51 (22.04, 25.56)	23.39 (20.77, 25.64)	.954
Hypertension	64 (35.6%)	50 (34.5%)	14 (40.0%)	.678
Diabetes mellitus	10 (5.6%)	9 (6.2%)	1 (2.9%)	.689
Hematuria, /μL	58.50 (21.00, 251.00)	60.00 (21.00, 260.00)	57.00 (22.00, 244.00)	.834
24-h UP, g/day	0.83 (0.40, 1.72)	0.83 (0.40, 1.57)	1.06 (0.38, 2.46)	.206
UACR, mg/g	396.80 (158.95, 939.00)	396.50 (143.20, 847.80)	403.10 (202.90, 1546.75)	.142
BUN, mmol/L	5.65 (4.40, 7.53)	5.80 (4.60, 7.50)	5.10 (4.25, 7.55)	.785
Scr, μmol/L	97.50 (80.00, 124.25)	100.00 (86.00, 123.00)	86.00 (66.00, 134.50)	.100
Cystatin C, mg/L	1.14 (0.92, 1.47)	1.14 (0.95, 1.47)	1.14 (0.84, 1.48)	.454
Baseline eGFR, mL/min/1.73 m²	75.97 (51.23, 97.97)	74.49 (52.00, 92.00)	89.97 (50.33, 113.53)	.167
UA, μmol/L	388.50 (327.25, 448.75)	392.00 (340.00, 448.00)	359.00 (303.50, 455.00)	.389
Serum IgA, g/L	3.01 (2.46, 3.85)	3.15 (2.50, 3.94)	2.79 (2.33, 3.43)	.135
Serum IgG, g/L	11.25 ± 2.88	11.39 ± 2.90	10.67 ± 2.76	.187
Complement C3, g/L	1.05 (0.92, 1.21)	1.05 (0.92, 1.22)	1.09 (0.95, 1.17)	.823
**CKD stage**				.060
CKD 1	58 (32.2%)	41 (28.3%)	17 (48.6%)	
CKD 2	63 (35.0%)	56 (38.6%)	7 (20.0%)	
CKD 3	49 (27.2%)	41 (28.3%)	8 (22.9%)	
CKD 4	10 (5.6%)	7 (4.8%)	3 (8.6%)	
HB, g/L	131.69 ± 19.25	132.66 ± 19.30	127.71 ± 18.78	.174
WBC, ×10⁹/L	6.68 (5.41, 8.31)	6.72 (5.40, 8.31)	6.64 (5.51, 8.05)	.714
PLT, ×10⁹/L	249.00 (213.75, 292.50)	244.00 (210.00, 289.00)	268.00 (231.50, 306.00)	.106
NLR	1.93 (1.38, 2.52)	1.91 (1.48, 2.46)	2.12 (1.35, 3.08)	.938
Albumin, g/L	41.00 (38.00, 45.00)	42.00 (39.00, 44.00)	41.00 (38.00, 45.00)	.499
Complement C4, g/L	0.25 (0.20, 0.31)	0.25 (0.20, 0.32)	0.24 (0.21, 0.30)	.874
TC, mmol/L	4.63 (4.01, 5.32)	4.63 (4.03, 5.31)	4.60 (3.88, 5.46)	.777
TG, mmol/L	1.34 (0.97, 1.90)	1.34 (1.00, 1.91)	1.35 (0.95, 1.89)	.99
LDL-C, mmol/L	2.65 (2.13, 3.29)	2.66 (2.12, 3.28)	2.60 (2.20, 3.31)	.848
HDL-C, mmol/L	1.18 (0.95, 1.43)	1.18 (0.94, 1.42)	1.20 (1.04, 1.43)	.666
**Renal biopsy pathology: MEST-C**				
M1	170 (94.4%)	135 (93.1%)	35 (100.0%)	.213
E1	52 (28.9%)	42 (29.0%)	10 (28.6%)	1
S1	108 (60.0%)	88 (60.7%)	20 (57.1%)	.848
**Oxford T grade**				.364
T0	129 (71.7%)	104 (71.7%)	25 (71.4%)	
T1	35 (19.4%)	30 (20.7%)	5 (14.3%)	
T2	16 (8.9%)	11 (7.6%)	5 (14.3%)	
**Oxford C grade**				.818
C0	96 (53.3%)	78 (53.8%)	18 (51.4%)	
C1	77 (42.8%)	62 (42.8%)	15 (42.9%)	
C2	7 (3.9%)	5 (3.4%)	2 (5.7%)	
**Renal biopsy pathology: Lee**				.29
Lee I–III	112 (62.2%)	87 (60.0%)	25 (71.4%)	
Lee IV–V	68 (37.8%)	58 (40.0%)	10 (28.6%)	
**Treatment**				
RAAS inhibitor	135 (75.0%)	111 (76.6%)	24 (68.6%)	.447
Glucocorticoid	64 (35.6%)	50 (34.5%)	14 (40.0%)	.678
Immunosuppressant	20 (11.1%)	18 (12.4%)	2 (5.7%)	.373
SGLT2 inhibitor	14 (7.8%)	12 (8.3%)	2 (5.7%)	1

Note. Normally distributed variables are shown as mean ± SD and compared by *t* test; non-normal variables as median (IQR) and compared by Mann–Whitney *U*. Categorical variables were compared by chi-square, continuity-corrected chi-square, or Fisher’s exact tests as appropriate. Cystatin C was available in 168 patients; available-case analysis was used. Rapid progression = eGFR slope < −5 mL/min/1.73 m²/year. BMI: body mass index; 24-h UP: 24-h urinary protein; UACR: urine albumin-to-creatinine ratio; Scr: serum creatinine; eGFR: estimated glomerular filtration rate; UA: uric acid; BUN: blood urea nitrogen; CKD: chronic kidney disease; HB: hemoglobin; WBC: white blood cell count; PLT: platelets; NLR: neutrophil-to-lymphocyte ratio; TC: total cholesterol; TG: triglycerides; M: mesangial hypercellularity; E: endocapillary hypercellularity; S: segmental glomerulosclerosis; T: tubular atrophy/interstitial fibrosis; C: crescents; RAAS: renin-angiotensin-aldosterone system; SGLT2: sodium-glucose cotransporter 2. IgG and HB are presented as mean ± SD; all other continuous variables are presented as median (IQR).

Oxford MEST-C findings included M1 in 170 patients (94.4%), E1 in 52 (28.9%), S1 in 108 (60.0%), T0 in 129 (71.7%), T1 in 35 (19.4%), T2 in 16 (8.9%), and C1/C2 in 84 (46.7%). Lee IV–V was present in 68 patients (37.8%). Treatment after biopsy included RAAS blockade in 135 patients (75.0%), corticosteroids in 64 (35.6%), and immunosuppressants in 20 (11.1%).

The median eGFR slope was −1.12 mL/min/1.73 m^2^/year (IQR −4.05 to 0.88); 35 patients (19.4%) were rapid progressors (Figure 1B). Slope distributions varied across baseline eGFR groups (Kruskal–Wallis *p* = .033, Figure 1C), whereas rapid-progression proportions did not differ significantly (*p* = .060, Figure 1D). These group comparisons were descriptive.

### Univariable analysis

Univariable linear regression identified associations of more negative eGFR slope with higher baseline eGFR (*β*= −0.032), ln(UACR + 1) (*β*= −0.579), ln(24h urine protein + 1) (*β*= −1.922), and M1 (*β*= −3.633; Table 2). The M1 estimate was imprecise because only 10 patients were M0. With Oxford T modeled categorically, neither T1 versus T0 (*β* = 1.336, *p* = .183) nor T2 versus T0 (*β*= −2.085, *p* = .135) was statistically significant; the global two-degree-of-freedom test for Oxford T was also not significant (*p* = .095).

**Table 2.** Univariable linear regression analysis of eGFR slope.

Variable	*β*	95%CI	*p*	*R*²
Age, years	0.006	(−0.057, 0.069)	.861	0.000
Male sex	1.186	(−0.391, 2.762)	.139	0.012
Hypertension	0.669	(−0.955, 2.293)	.417	0.004
Diabetes mellitus	−0.425	(−3.825, 2.974)	.805	0.000
Baseline eGFR	−0.032	(−0.058, −0.007)	.014*	0.033
Scr, μmol/L	0.008	(−0.007, 0.023)	.277	0.007
BUN, mmol/L	0.122	(−0.153, 0.397)	.383	0.004
UA, μmol/L	0.004	(−0.003, 0.012)	.250	0.007
ln(24-h UP+1)	−1.922	(−3.455, −0.389)	.014*	0.033
ln(UACR+1)	−0.579	(−1.034, −0.124)	.013*	0.034
Hematuria, /μL	0.001	(0.000, 0.003)	.101	0.015
Albumin, g/L	0.109	(−0.028, 0.247)	.119	0.014
IgA, g/L	0.299	(−0.209, 0.806)	.247	0.008
IgG, g/L	0.360	(0.094, 0.626)	.008*	0.039
Complement C3, g/L	1.826	(−1.678, 5.329)	.305	0.006
Cystatin C, mg/L	0.628	(−0.667, 1.922)	.340	0.005
HB, g/L	0.002	(−0.039, 0.042)	.940	0.000
WBC, ×10⁹/L	0.315	(−0.031, 0.661)	.074	0.018
PLT, ×10⁹/L	−0.001	(−0.012, 0.011)	.910	0.000
NLR	0.626	(0.073, 1.179)	.027*	0.027
M1	−3.633	(−6.990, −0.276)	0.034*	0.025
E1	0.498	(−1.219, 2.215)	0.568	0.002
S1	0.554	(−1.034, 2.142)	0.492	0.003
Oxford T1 vs T0	1.336	(−0.635, 3.307)	0.183	0.026
Oxford T2 vs T0	−2.085	(−4.826, 0.655)	0.135	0.026
C1/C2	−0.023	(−1.584, 1.538)	0.977	0.000
Lee IV-V	1.180	(−0.416, 2.777)	0.146	0.012
RAAS inhibitor	−0.065	(−1.863, 1.734)	0.943	0.000
Glucocorticoid	0.714	(−0.909, 2.338)	0.386	0.004
SGLT2 inhibitor	1.241	(−1.661, 4.143)	0.400	0.004
Immunosuppressant	1.973	(−0.488, 4.434)	0.115	0.014

Note: Negative β indicates an association with a more negative eGFR slope. Natural logarithms were used for transformed UACR and 24-h urinary protein. Oxford T contrasts use T0 as the reference; the global two-degree-of-freedom *p* value for Oxford T was 0.095, and the *R*² shown for both category rows is from the joint categorical-T model. M1 estimates are unstable because only 10 patients were M0. 24-h UP, 24-h urinary protein; UACR: urine albumin-to-creatinine ratio; Scr: serum creatinine; eGFR: estimated glomerular filtration rate; WBC: white blood cell count; NLR: neutrophil-to-lymphocyte ratio; RAAS: renin–angiotensin–aldosterone system; SGLT2: sodium-glucose cotransporter 2.

*p < 0.05.

### Multivariable analysis

The *post hoc* primary model explained 15.9% of eGFR-slope variance (adjusted *R*^2^=0.130; *F* = 5.46; *p* < .001; Table 3). Higher ln(UACR + 1) was associated with faster decline (*β*= −0.612, 95%CI −1.073 to −0.150; *p* = .010). The global two-degree-of-freedom test for categorical Oxford T was significant (*p* = .034). T2 versus T0 was associated with a 3.87 mL/min/1.73 m^2^/year more negative slope (*β*= −3.870, 95%CI −7.087 to −0.654; *p* = .019), whereas T1 did not differ from T0 (*β* = 0.041, *p* = .970). All VIFs were <1.7. An increase in UACR from 300 to 600 mg/g corresponded to an estimated 0.42 mL/min/1.73 m^2^/year more negative slope, and an increase from 300 to 1000 mg/g corresponded to an estimated 0.73 mL/min/1.73 m^2^/year more negative slope.

**Table 3.** *Post hoc* exploratory multivariable linear regression model for eGFR slope.

Variable	β (95%CI)	*p* Value	VIF	Std *β*
Baseline eGFR, mL/min/1.73 m²	−0.054 (−0.085 to −0.022)	<0.001	1.65	−0.305
ln(UACR+1), mg/g	−0.612 (−1.073 to −0.150)	0.010	1.15	−0.195
Hematuria, /μL	0.002 (0.000 to 0.003)	0.025	1.03	0.160
WBC, ×10⁹/L	0.365 (0.038 to 0.693)	0.029	1.02	0.155
Oxford T1 vs T0	0.041 (−2.125 to 2.207)	0.970	1.40	0.003
Oxford T2 vs T0	−3.870 (−7.087 to −0.654)	0.019	1.60	−0.209

Note: Model statistics: *R*²=0.159; adjusted *R*²=0.130; *F* = 5.46; *p*(model)<.001. The model was developed *post hoc* using prior literature, clinical judgment, data completeness, and parsimony. Natural logarithms were used for transformed UACR. Oxford T was categorical (T0 reference), with a global two-degree-of-freedom *p* value of .034. Lee grade was omitted because of overlap with Oxford T; treatment variables were omitted because exposure began after biopsy and overlapped the outcome window. Negative *β* indicates an association with a more negative slope. Hematuria and WBC count carry positive coefficients; these should not be interpreted as biologically protective against disease progression, but rather as likely reflections of mathematical coupling, regression to the mean, or residual confounding. UACR: urine albumin-to-creatinine ratio; WBC: white blood cell count; VIF: variance inflation factor; Std β: standardized regression coefficient. Caution: The negative coefficient for baseline eGFR reflects mathematical coupling with the calculated slope and regression to the mean; it should not be interpreted as a biological risk factor for faster decline.

Baseline eGFR, hematuria, and WBC count retained statistical significance, yet their coefficients probably reflect mathematical coupling, regression to the mean, or residual confounding rather than genuine biological protection. We do not treat these as causal. When Oxford T was entered as an ordinal variable, it fell short of significance (*β*= −1.420, *p* = .067). Lee IV–V, when substituted for Oxford T, showed no independent association with slope (*β* = 1.361, *p* = .166). Fitting both Lee grade and Oxford T together produced unstable coefficients (Supplementary Table S4).

### Exploratory discrimination of rapid progression

Exploratory apparent AUCs were 0.623 (95%CI 0.524–0.715) for the clinical model, 0.661 (0.567–0.754) for the pathological model, 0.724 (0.634–0.814) for the combined model, and 0.755 (0.662–0.841) for the full model. Optimism-corrected AUCs were lower at 0.571, 0.596, 0.642, and 0.660, respectively. (Supplementary Figure S1 and Supplementary Table S7).

### Sensitivity analyses

Details of the sensitivity analyses appear in Supplementary Tables S2–S6. HC3 robust standard errors preserved the negative associations for ln(UACR + 1) (*p* = .003) and T2 versus T0 (*p* = .039). After dropping 13 influential observations, the corresponding estimates were *β*= −0.493 (*p* = .013) and *β*= −3.007 (*p* = .036). Weighting by measurement count also left both estimates negative. In the restricted subset with ≥5 measurements, however, both estimates lost magnitude and precision, with T2 particularly affected. Bootstrap percentile intervals excluded zero for both variables, yet optimism correction reduced *R*^2^ to 0.104.

With baseline eGFR removed, ln(UACR + 1) stayed significantly associated with steeper decline (*β*= −0.708, *p* = .004); the T2 coefficient, by contrast, shrank toward null (*β*= −0.834, *p* = .553). In the subset without immunosuppressant users, both ln(UACR + 1) (*β*= −0.725, *p* = .003) and T2 (*β*= −4.161, *p* = .019) again showed negative estimates. Across these analyses, albuminuria proved the more robust marker. The T2 association was less stable and should be treated as hypothesis-generating.

## Discussion

In this retrospective cohort, baseline albuminuria proved the most consistent adverse marker of eGFR slope. T2 predicted faster decline in the primary model and several sensitivity analyses, yet the estimate rested on only 16 patients and lost precision in some restricted specifications. We do not treat baseline eGFR, hematuria, or WBC coefficients as causal or protective.

Proteinuria has long been recognized as a key determinant of kidney-function loss in IgAN [16,17]. The link between higher ln(UACR + 1) and steeper decline held directionally across the main sensitivity analyses. Raising UACR from 300 to 600 mg/g translated into roughly 0.42 mL/min/1.73 m^2^/year faster annual loss; raising it from 300 to 1,000 mg/g translated into roughly 0.73 mL/min/1.73 m^2^/year faster loss. Reducing proteinuria therefore remains a central therapeutic goal [18].

Oxford T2 – severe tubular atrophy/interstitial fibrosis – was associated with an additional 3.87 mL/min/1.73 m^2^/year decline relative to T0 in the primary model [19,20]. However, this estimate derived from only 16 patients (8.9%) and was not robust to sample restrictions: in the ≥5-measurement subset, the coefficient attenuated to −1.556 and lost statistical significance (*p* = .246). T1 did not differ from T0, and the estimate also shrank when baseline eGFR was omitted. We therefore do not infer a simple linear per-grade effect; the T2 finding is tentative and exploratory.

Lee IV–V showed no independent link to slope when it replaced Oxford T in an otherwise comparable model. The two classifications were strongly correlated (Spearman *r* = 0.706), and entering both produced a positive Lee coefficient alongside a more negative T2 coefficient. This pattern reflects coefficient suppression between correlated severity markers; it does not imply that advanced Lee grade protects kidney function. The primary model retained Oxford T and dropped Lee grade.

The negative baseline-eGFR coefficient demands methodological caution, as baseline eGFR enters both the predictor set and the slope calculation itself. Regression to the mean, greater room for absolute decline from higher starting values, and imprecision in the high-eGFR range all likely play a part. Positive hematuria and WBC coefficients probably stem from residual confounding, skewed measurements, treatment selection, or model dependence rather than from true biological protection.

We excluded post-biopsy treatment from the primary model. Because therapy began within 6 months of biopsy, the exposure window overlapped with slope follow-up, opening the analysis to time-dependent bias, immortal-time bias, reverse causation, and confounding by indication. The dataset also lacked time-varying data on drug dose, duration, adherence, or switches. We make no treatment-effect claims from these observational data; randomized trials or appropriately time-dependent analyses would be required [21].

We kept the discrimination analysis only as a supplementary exploratory description. Apparent AUCs rose with model complexity, yet confidence intervals overlapped and optimism correction pulled all estimates down to 0.571–0.660. Whether such models deliver genuine incremental predictive value in clinical practice is unclear. After optimism correction, the primary linear model explained only 10.4% of the variance. Baseline variables leave roughly 90% of eGFR trajectory variation unaccounted for, limiting the model’s value for individual prognostic counseling. Dynamic approaches may improve IgAN risk assessment, but they too require rigorous external validation [22,23].

This study has several important limitations. The retrospective single-center design and the requirement for ≥3 years of follow-up with ≥3 valid eGFR values may have excluded patients with early kidney failure, early loss to follow-up, or infrequent monitoring, limiting generalizability. Although eGFR was recorded at nominal 6-month landmarks and AKI values were excluded, missing measurements were common at later time points. Patient-level linear slopes ignore within-patient error and nonlinear trajectories. Exact encounter dates were unavailable, precluding a random-slope mixed-effects model; a mixed-effects analysis using nominal landmarks would still retain timing assumptions. Prospective studies with exact visit dates should adopt mixed-effects or spline-based trajectory models. The cohort was modest, with only 35 rapid progressors and 16 T2 cases. The model was built *post hoc*, treatment exposure was time-dependent, and neither the regression nor the ROC findings were externally validated.

Future research should employ prospectively scheduled visits with exact dates, repeated-measures mixed-effects or spline models, time-updated treatment analyses, prespecified model development, and external validation [24].

In conclusion, baseline albuminuria was the most consistent marker of faster eGFR decline in this cohort. Oxford T2 also predicted a steeper slope, but the small subgroup and variable sensitivity-analysis results make this finding tentative. The remaining adjusted associations, treatment effects, and rapid-progression discrimination results are exploratory. These data are hypothesis-generating and should not be used for individual prediction or treatment decisions until prospective external validation is available.

## Supplementary Material

IgAN R2 Supplementary Material.docx

Supplementary Figure S1 ROC R2.tif

## Data Availability

Available from the corresponding author upon reasonable request, subject to institutional and ethical approvals. Public archiving is restricted because the dataset contains potentially identifiable patient information governed by the Ethics Committee of Zhongshan Hospital (Xiamen), Fudan University, and applicable Chinese personal health data protection regulations prohibit open deposition of individual-level clinical records.
