# Supplementary material for: Clinical and pathological factors associated with eGFR slope in IgA nephropathy: a single-center retrospective cohort study
Source: Ren Fail. 2026 Oct 7;48(1):2732704. doi: 10.1080/0886022X.2026.2732704 (PMC13647398; doi:10.1080/0886022X.2026.2732704)
Supplement: Supplementary file 1 [file IRNF_A_2732704_SM7099.docx]

Supplementary Material

**Supplementary Table S1. eGFR measurement schedule, availability, and frequency**

| **Measurement/frequency** | **Available, n/N (%)** | **Missing, n** | **Analysis handling** |
| --- | --- | --- | --- |
| Baseline | 180/180 (100.0%) | 0 | Included in slope and primary model |
| 6 months | 158/180 (87.8%) | 22 | Included in slope when available; not imputed |
| 12 months | 151/180 (83.9%) | 29 | Included in slope when available; not imputed |
| 18 months | 142/180 (78.9%) | 38 | Included in slope when available; not imputed |
| 24 months | 130/180 (72.2%) | 50 | Included in slope when available; not imputed |
| 30 months | 110/180 (61.1%) | 70 | Included in slope when available; not imputed |
| 36 months | 107/180 (59.4%) | 73 | Included in slope when available; not imputed |
| Patients contributing 3 total eGFR values | 30/180 (16.7%) | — | Measurement-frequency distribution |
| Patients contributing 4 total eGFR values | 26/180 (14.4%) | — | Measurement-frequency distribution |
| Patients contributing 5 total eGFR values | 28/180 (15.6%) | — | Measurement-frequency distribution |
| Patients contributing 6 total eGFR values | 28/180 (15.6%) | — | Measurement-frequency distribution |
| Patients contributing 7 total eGFR values | 68/180 (37.8%) | — | Measurement-frequency distribution |

Note: eGFR values correspond to baseline and nominal 6-, 12-, 18-, 24-, 30-, and 36-month follow-up assessments. Patients contributed a median of 6 measurements (IQR 4–7; range 3–7). The last available eGFR landmark was 36 months (IQR 24–36). Values recorded during documented acute kidney injury episodes had been excluded before slope calculation. Baseline UACR, 24-hour urine protein, hematuria, WBC, Oxford MEST-C, and Lee grade were complete.

**Supplementary Table S2. Primary model with conventional and HC3 inference**

| **Inference** | **Variable** | **β** | **95% CI** | **P value** |
| --- | --- | --- | --- | --- |
| Conventional OLS | Baseline eGFR | -0.054 | -0.085 to -0.022 | <0.001 |
| Conventional OLS | ln(UACR+1) | -0.612 | -1.073 to -0.150 | 0.010 |
| Conventional OLS | Hematuria | 0.002 | 0.000 to 0.003 | 0.025 |
| Conventional OLS | WBC | 0.365 | 0.038 to 0.693 | 0.029 |
| Conventional OLS | T1 vs T0 | 0.041 | -2.125 to 2.207 | 0.970 |
| Conventional OLS | T2 vs T0 | -3.870 | -7.087 to -0.654 | 0.019 |
| HC3 robust inference | Baseline eGFR | -0.054 | -0.088 to -0.019 | 0.003 |
| HC3 robust inference | ln(UACR+1) | -0.612 | -1.014 to -0.209 | 0.003 |
| HC3 robust inference | Hematuria | 0.002 | 0.001 to 0.003 | 0.003 |
| HC3 robust inference | WBC | 0.365 | 0.005 to 0.726 | 0.047 |
| HC3 robust inference | T1 vs T0 | 0.041 | -2.094 to 2.176 | 0.970 |
| HC3 robust inference | T2 vs T0 | -3.870 | -7.544 to -0.196 | 0.039 |

Note: HC3 uses the same point estimates with heteroscedasticity-consistent standard errors. Natural logarithms were used for transformed UACR. T0 is the reference category; the global two-degree-of-freedom P value for Oxford T in the primary model was 0.034.

**Supplementary Table S3. Sensitivity analyses of slope estimation and cohort restrictions**

| **Scenario** | **N** | **R²** | **Variable** | **β** | **95% CI** | **P value** |
| --- | --- | --- | --- | --- | --- | --- |
| Weighted by number of eGFR values | 180 | 0.117 | Baseline eGFR | -0.044 | -0.073 to -0.016 | 0.002 |
| Weighted by number of eGFR values | 180 | 0.117 | ln(UACR+1) | -0.491 | -0.919 to -0.063 | 0.025 |
| Weighted by number of eGFR values | 180 | 0.117 | Hematuria | 0.001 | 0.000 to 0.003 | 0.032 |
| Weighted by number of eGFR values | 180 | 0.117 | WBC | 0.251 | -0.044 to 0.546 | 0.095 |
| Weighted by number of eGFR values | 180 | 0.117 | T1 vs T0 | -0.241 | -2.159 to 1.677 | 0.805 |
| Weighted by number of eGFR values | 180 | 0.117 | T2 vs T0 | -2.881 | -5.694 to -0.068 | 0.045 |
| Restricted to ≥5 eGFR values | 124 | 0.056 | Baseline eGFR | -0.027 | -0.055 to 0.001 | 0.056 |
| Restricted to ≥5 eGFR values | 124 | 0.056 | ln(UACR+1) | -0.264 | -0.671 to 0.143 | 0.202 |
| Restricted to ≥5 eGFR values | 124 | 0.056 | Hematuria | 0.001 | -0.000 to 0.003 | 0.168 |
| Restricted to ≥5 eGFR values | 124 | 0.056 | WBC | 0.088 | -0.197 to 0.373 | 0.543 |
| Restricted to ≥5 eGFR values | 124 | 0.056 | T1 vs T0 | -0.653 | -2.415 to 1.109 | 0.464 |
| Restricted to ≥5 eGFR values | 124 | 0.056 | T2 vs T0 | -1.556 | -4.196 to 1.084 | 0.246 |
| Baseline eGFR omitted | 180 | 0.103 | ln(UACR+1) | -0.708 | -1.180 to -0.235 | 0.004 |
| Baseline eGFR omitted | 180 | 0.103 | Hematuria | 0.001 | -0.000 to 0.003 | 0.053 |
| Baseline eGFR omitted | 180 | 0.103 | WBC | 0.382 | 0.045 to 0.720 | 0.027 |
| Baseline eGFR omitted | 180 | 0.103 | T1 vs T0 | 1.848 | -0.105 to 3.800 | 0.064 |
| Baseline eGFR omitted | 180 | 0.103 | T2 vs T0 | -0.834 | -3.607 to 1.939 | 0.553 |
| Cook's distance ≤4/n | 167 | 0.110 | Baseline eGFR | -0.034 | -0.061 to -0.007 | 0.015 |
| Cook's distance ≤4/n | 167 | 0.110 | ln(UACR+1) | -0.493 | -0.878 to -0.107 | 0.013 |
| Cook's distance ≤4/n | 167 | 0.110 | Hematuria | 0.001 | -0.001 to 0.002 | 0.335 |
| Cook's distance ≤4/n | 167 | 0.110 | WBC | 0.313 | 0.009 to 0.618 | 0.044 |
| Cook's distance ≤4/n | 167 | 0.110 | T1 vs T0 | -0.141 | -1.993 to 1.712 | 0.881 |
| Cook's distance ≤4/n | 167 | 0.110 | T2 vs T0 | -3.007 | -5.816 to -0.197 | 0.036 |
| Immunosuppressant users excluded | 160 | 0.174 | Baseline eGFR | -0.042 | -0.076 to -0.009 | 0.012 |
| Immunosuppressant users excluded | 160 | 0.174 | ln(UACR+1) | -0.725 | -1.193 to -0.258 | 0.003 |
| Immunosuppressant users excluded | 160 | 0.174 | Hematuria | 0.002 | 0.000 to 0.003 | 0.025 |
| Immunosuppressant users excluded | 160 | 0.174 | WBC | 0.322 | -0.013 to 0.656 | 0.060 |
| Immunosuppressant users excluded | 160 | 0.174 | T1 vs T0 | 0.666 | -1.582 to 2.913 | 0.559 |
| Immunosuppressant users excluded | 160 | 0.174 | T2 vs T0 | -4.161 | -7.629 to -0.692 | 0.019 |

Note: The ≥5-measurement restriction reduced the sample from 180 to 124. Thirteen observations exceeded Cook's distance 4/n.

**Supplementary Table S4. Alternative pathology model specifications**

| **Specification** | **N** | **R²** | **Variable** | **β** | **95% CI** | **P value** |
| --- | --- | --- | --- | --- | --- | --- |
| Oxford T ordinal | 180 | 0.142 | Baseline eGFR | -0.056 | -0.087 to -0.025 | <0.001 |
| Oxford T ordinal | 180 | 0.142 | ln(UACR+1) | -0.603 | -1.068 to -0.138 | 0.011 |
| Oxford T ordinal | 180 | 0.142 | Hematuria | 0.002 | 0.000 to 0.003 | 0.020 |
| Oxford T ordinal | 180 | 0.142 | WBC | 0.373 | 0.043 to 0.703 | 0.027 |
| Oxford T ordinal | 180 | 0.142 | Oxford T, ordinal | -1.420 | -2.943 to 0.103 | 0.067 |
| Lee IV–V replacing Oxford T | 180 | 0.135 | Baseline eGFR | -0.028 | -0.056 to 0.001 | 0.062 |
| Lee IV–V replacing Oxford T | 180 | 0.135 | ln(UACR+1) | -0.875 | -1.356 to -0.394 | <0.001 |
| Lee IV–V replacing Oxford T | 180 | 0.135 | Hematuria | 0.002 | 0.000 to 0.003 | 0.017 |
| Lee IV–V replacing Oxford T | 180 | 0.135 | WBC | 0.360 | 0.028 to 0.692 | 0.034 |
| Lee IV–V replacing Oxford T | 180 | 0.135 | Lee IV–V | 1.361 | -0.568 to 3.291 | 0.166 |
| T1/T2 binary | 180 | 0.128 | Baseline eGFR | -0.044 | -0.075 to -0.014 | 0.005 |
| T1/T2 binary | 180 | 0.128 | ln(UACR+1) | -0.696 | -1.160 to -0.232 | 0.004 |
| T1/T2 binary | 180 | 0.128 | Hematuria | 0.002 | 0.000 to 0.003 | 0.018 |
| T1/T2 binary | 180 | 0.128 | WBC | 0.376 | 0.044 to 0.709 | 0.027 |
| T1/T2 binary | 180 | 0.128 | T1/T2 vs T0 | -0.724 | -2.838 to 1.391 | 0.500 |
| Categorical T plus Lee IV–V | 180 | 0.181 | Baseline eGFR | -0.047 | -0.078 to -0.015 | 0.004 |
| Categorical T plus Lee IV–V | 180 | 0.181 | ln(UACR+1) | -0.757 | -1.234 to -0.280 | 0.002 |
| Categorical T plus Lee IV–V | 180 | 0.181 | Hematuria | 0.002 | 0.000 to 0.003 | 0.024 |
| Categorical T plus Lee IV–V | 180 | 0.181 | WBC | 0.339 | 0.014 to 0.664 | 0.041 |
| Categorical T plus Lee IV–V | 180 | 0.181 | T1 vs T0 | -1.334 | -3.831 to 1.163 | 0.293 |
| Categorical T plus Lee IV–V | 180 | 0.181 | T2 vs T0 | -5.265 | -8.704 to -1.826 | 0.003 |
| Categorical T plus Lee IV–V | 180 | 0.181 | Lee IV–V | 2.392 | 0.167 to 4.618 | 0.035 |

Note: All models additionally included baseline eGFR, ln(UACR+1), hematuria, and WBC unless the variable shown was the pathology term. Oxford T and Lee IV–V were strongly correlated (Spearman r=0.706). Their simultaneous inclusion generated a positive Lee coefficient and a more negative T2 coefficient, consistent with suppression/unstable partitioning; the primary model therefore used categorical Oxford T without Lee grade.

**Supplementary Table S5. Regression diagnostics and influential-observation assessment**

| **Assessment** | **Statistic** | **Interpretation/sensitivity result** |
| --- | --- | --- |
| Shapiro–Wilk residual normality | W=0.943 | P<0.001; residuals departed from normality |
| Breusch–Pagan heteroscedasticity | LM=4.634, df=6 | P=0.592; no evidence of heteroscedasticity |
| Cook's distance | Threshold 4/n=0.0222 | 13 observations exceeded threshold; maximum=0.108 |
| Collinearity | Maximum VIF=1.65 | No material collinearity in the primary categorical-T model |
| Influence sensitivity | N=167 | ln(UACR+1): β=−0.493, P=0.013; T2 vs T0: β=−3.007, P=0.036 |

Note: Because residual normality was imperfect, conventional results were compared with HC3 inference and nonparametric bootstrap intervals. Inferences for ln(UACR+1) and T2 were retained in those analyses.

**Supplementary Table S6. Bootstrap validation of the fixed post hoc model (2,000 resamples)**

| **Variable** | **Primary β** | **Bootstrap 95% percentile CI** | **Sign consistency** |
| --- | --- | --- | --- |
| Baseline eGFR | -0.054 | -0.085 to -0.019 | 99.8% |
| ln(UACR+1) | -0.612 | -1.005 to -0.238 | 100.0% |
| Hematuria | 0.002 | 0.000 to 0.002 | 97.8% |
| WBC | 0.365 | 0.098 to 0.748 | 99.7% |
| T1 vs T0 | 0.041 | -1.995 to 2.230 | 51.4% |
| T2 vs T0 | -3.870 | -7.558 to -0.377 | 98.4% |

Note: Patient-level nonparametric bootstrap resampling refitted the unchanged six-parameter predictor set in 2,000 resamples (seed 20260819). This procedure assessed the internal stability of the fixed model, not the post hoc selection process. Apparent R²=0.159; mean optimism=0.055; optimism-corrected R²=0.104.

**Supplementary Table S7. Exploratory ROC analysis with confidence intervals and optimism correction**

| **Model** | **Predictors, n** | **Apparent AUC** | **95% bootstrap CI** | **Mean optimism** | **Corrected AUC** |
| --- | --- | --- | --- | --- | --- |
| Clinical | 4 | 0.623 | 0.524 to 0.715 | 0.052 | 0.571 |
| Pathological | 6 | 0.661 | 0.567 to 0.754 | 0.065 | 0.596 |
| Combined | 10 | 0.724 | 0.634 to 0.814 | 0.081 | 0.642 |
| Full | 14 | 0.755 | 0.662 to 0.841 | 0.094 | 0.660 |

Note: The binary outcome comprised 35 rapid progressors and 145 non-rapid progressors. Predictors were standardized and fitted using L2-penalized logistic regression (C=1.0). Confidence intervals used 2,000 stratified bootstrap samples of fixed predictions; optimism used 1,000 stratified bootstrap refits.
